# Combination Treatments with the PKC Inhibitor, Enzastaurin, Enhance the Cytotoxicity of the Anti-Mesothelin Immunotoxin, SS1P

**DOI:** 10.1371/journal.pone.0075576

**Published:** 2013-10-09

**Authors:** Abid R. Mattoo, Ira Pastan, David FitzGerald

**Affiliations:** Laboratory of Molecular Biology, Center for Cancer Research, National Cancer Institute, National Institutes of Health, Bethesda, Maryland, United States of America; Wayne State University School of Medicine, United States of America

## Abstract

Activated protein kinase C (PKC) contributes to tumor survival and proliferation, provoking the development of inhibitory agents as potential cancer therapeutics. Immunotoxins are antibody-based recombinant proteins that employ antibody fragments for cancer targeting and bacterial toxins as the cytotoxic agent. Pseudomonas exotoxin-based immunotoxins act via the ADP-ribosylation of EF2 leading to the enzymatic inhibition of protein synthesis. Combining PKC inhibitors with the immunotoxin SS1P, targeted to surface mesothelin, was undertaken to explore possible therapeutic strategies. Enzastaurin but not two other PKC inhibitors combined with SS1P to produce synergistic cell death via apoptosis. Mechanistic insights of the synergistic killing centered on the complete loss of the prosurvival Bcl2 protein, Mcl-1, the loss of AKT and the activation of caspase 3/7. Synergy was most evident when cells exhibited resistance to the immunotoxin alone. Further, because PKC inhibition by itself was not sufficient to enhance SS1P action, enzastaurin must target other kinases that are involved in the immunotoxin pathway.

## Introduction

Protein Kinase C (PKC) enzymes contribute to growth, survival and angiogenesis, all features that are frequently up-regulated in cancer [1]. Therefore, PKCs represent a potentially important target for pharmacological intervention [2]. In mammals there are eight homologous isoforms including four ‘conventional’ and four ‘novel’ enzymes. These serine-threonine kinases are configured with N-terminal regulatory domains and a C-terminal enzymatic domain. Activation, which involves relocation from the cytosol to a membrane, is via diacylglycerol (DAG), calcium or various phorbol esters. When targeting PKCs, inhibition of specific isoforms is complicated by the close similarity of C-terminal domains. Consequently, low molecular weight inhibitors that target a specific enzymatic domain are still likely to exhibit a range of inhibitory actions against most family members. This leads to an empirical approach whereby inhibitors are tested for effectiveness based on biochemical or phenotypic outcomes. Here we survey three known PKC inhibitors, enzastaurin [3], Go6976 [4] and sotrastaurin [5] and investigate their ability to enhance the killing of an immunotoxin directed to the cell surface antigen, mesothelin.

Because most antibodies do not exhibit cell-killing activity in an unmodified form, they are frequently joined to toxic molecules to increase killing activity [6] [7]. One modification is the fusing of a bacterial toxin to the Fv fragment of a cell-targeting antibody to generate a recombinant immunotoxin [8] [9]. Recombinant immunotoxins are designed so that the antibody fragment binds a surface antigen and the toxin, after internalization, kills the cell. When targeting cancer cells, the strategy is to target receptors or antigens that are not expressed on vital normal tissues but are expressed uniformly on the malignancy [10]. The advantage of using bacterial toxins resides in the potency of the enzyme domain associated with the toxin. In the case of Pseudomonas exotoxin (PE), this domain functions as an ADP-ribosyl transferase that modifies elongation factor 2 (EF2) leading to inhibition of protein synthesis [11]. Further, a particular advantage of using an agent that inhibits protein synthesis is the negation of adaptive survival pathways that rely on gene expression and the synthesis of new protein products such as chaperones or survival factors [12]. Until recently, the inhibition of protein synthesis by bacterial toxins was thought to be a lethal event [13] [14], [15], [16]. For reasons that are not fully understood, some toxin-treated mammalian cells appear to survive toxin treatment. Thus, we have begun to investigate agents that increase cell killing and therefore might be useful in combination with immunotoxins.

The immunotoxin, SS1P, is targeted to surface mesothelin which is up-regulated on a number of epithelial cancers including pancreatic, lung, ovarian and mesotheliomas [17], [18], [19], [20]. Expression of mesothelin on normal tissues is limited to the cells lining the peritoneal cavity and pericardium. In clinical trials treating human epithelial cancers, SS1P has not demonstrated consistent objective responses when administered as single agent [19], [21]. Also there has been a strong immune response to the toxin portion of the immunotoxin [19], [21]. Thus, immunotoxins suffer from two potential problems, one is an immunogenic response by the host and the other is a failure to kill sufficient target cells to achieve complete remissions. The former is being addressed by removing prominent B and T cell epitopes [22], [23], [24], [25]. To address the latter, we and others are investigating agents to be used in combination with immunotoxins to enhance killing action [13], [26], [27], [28], [29], [30].

To investigate new approaches for enhancing immunotoxin action, we reasoned that kinase inhibitors might be a particularly apt choice because they target survival pathways and because they do not require the expression of new gene products to be effective. We surveyed three inhibitors of PKC and report that enzastaurin exhibited immunotoxin enhancing action while the other two did not. Enhancement was noted with SS1P and to a lesser extent with a model immunotoxin targeting the transferrin receptor. However, there was no enhancement of other agents that inhibit protein synthesis such as diphtheria toxin and cycloheximide, suggesting a specific action on the PE-based immunotoxin pathway.

## Materials and Methods

### Cytotoxic Agents

Enzastaurin was purchased from Selleck Chemicals LLC, dissolved in DMSO at 10 mmol/L and stored frozen at −80°C. Sotrastaurin was purchased from Axon Medchem, dissolved in DMSO at 10 mm/L and stored at −20°C. Go6976 was purchased from EMD Millipore, dissolved in DMSO at 5 mmol/L and stored at −20°C. SS1P and HB21-PE40 were produced recombinantly in *Escherichia coli* as described previously. Cycloheximide was purchased from Sigma.

### Cell Lines

The KB3-1 cells (cervical cancer cells, called ‘KB cells’ below) were obtained from Dr Michael Gottesman, NCI and grown in DMEM plus 10% FBS [31]. KLM1 cells (pancreatic cancer line) were obtained from the RIKEN cell bank via Udo Rudloff, NCI. HAY cell line (mesothelioma) was obtained from Dr Raffit Hassan [32], NCI and grown RPMI-1640 medium containing 2 mM L-glutamine and 1 mM sodium pyruvate.

### Assays

CellTiter-Glo (Promega) and Caspase-Glo (Promega) measured cytotoxic activity and were used according to the directions supplied by the manufacturers. Routinely, cells were incubated for 48 hr prior to the addition of CellTiter-Glo reagent or overnight when measuring caspase activity. To stain treated cells, methylene blue (0.5% w/v) in 50% (v/v) methanol/water was added for ∼15 min. Treated cells were assayed for inhibition of protein synthesis by the addition of ^3^H-leucine (2 µCi/ml) for 2 hrs in 96-well plates. Cells were collected on filter mats and samples counted using a Wallac Beta plate reader.

### Western Blot Analysis

Immunotoxin-treated cells in the presence or absence of Enzastaurin, Sotrastaurin and Go6976 were washed with PBS, and then solubilized with RIPA buffer containing both protease and phosphatase inhibitors. Precast NU-PAGE 8–16% gels were used to separate cell lysates. Lysates transferred to nitrocellulose membranes were probed with rabbit anti-Mcl-1 (Santa Cruz Biotechnology, Catalog No sc-819), rabbit anti-Akt (Cell Signalling Technology, Catalog No 9272) anti-β-actin (BD-Transduction Labs, Catalog No. 612656). The primary antibody was detected with goat anti-rabbit or anti-mouse HRP (Jackson Immunoresearch).

## Results

### Survey of PKC Inhibitors

**Figure 1:**
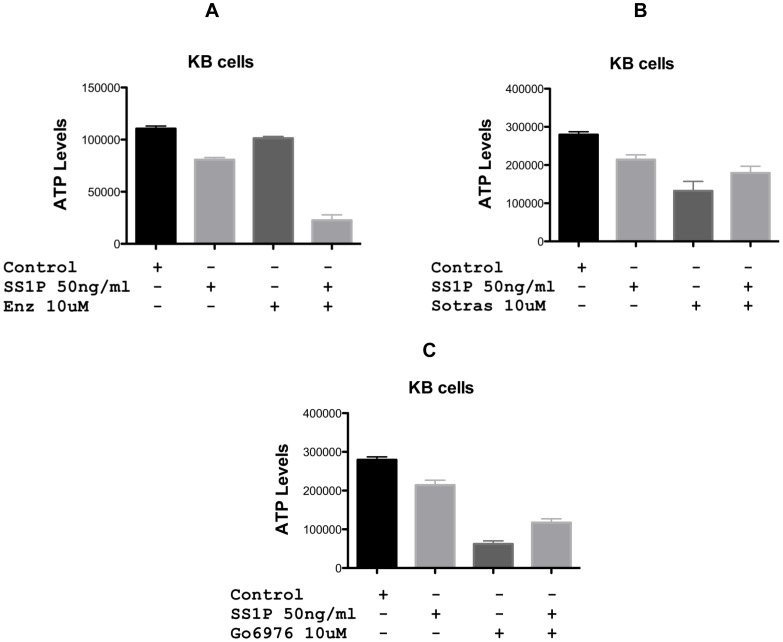
Cytotoxic activity of the anti-mesothelin immunotoxin, SS1P, in combination with PKC inhibitors on KB cells. PKC inhibitors (Enzastaurin, Sortastaurin or Go6976) at 10 uM were added in combination with SS1P (50 ng/ml) to KB cells and viability assessed using the CellTiter-Glo® Viability Assay after 48 hrs. Enz = enzastaurin, Sotras = sotrastaurin.

Because combination therapies are frequently more effective in cancer treatment than single agents, we evaluated three PKC inhibitors in combination with SS1P, an immunotoxin targeted to surface mesothelin. Enzastaurin, sotrastaurin and Go6976 at 5 (not shown) and 10 uM were incubated with KB cells either alone or in combination with SS1P. After 48 hr, cytotoxicity was determined using the CellTiterGlo assay. By itself, enzastaurin showed only modest inhibitory activity at 10 uM. However, when added in combination, the toxicity of SS1P was greatly enhanced (Fig. 1A). At 50 ng/ml SS1P alone caused no more than a 20% reduction in growth. The combination of SS1P and 10 uM of enzastaurin caused a ∼80% reduction (Fig. 1 A). As single agents, sotrastaurin (10 uM) and G06976 (10 uM) exhibited a 50–70% reduction in growth (Figs. 1 B and C). However, combinations of either compound with SS1P were apparently antagonistic. Specifically, the activity of both PKC inhibitors was reduced when co-incubated with SS1P. Because of the potent enhancing action of enzastaurin, it was studied more extensively.

### Enzastaurin Enhancement of SS1P Targeted to Mesothelin-positive Cells

**Figure 2:**
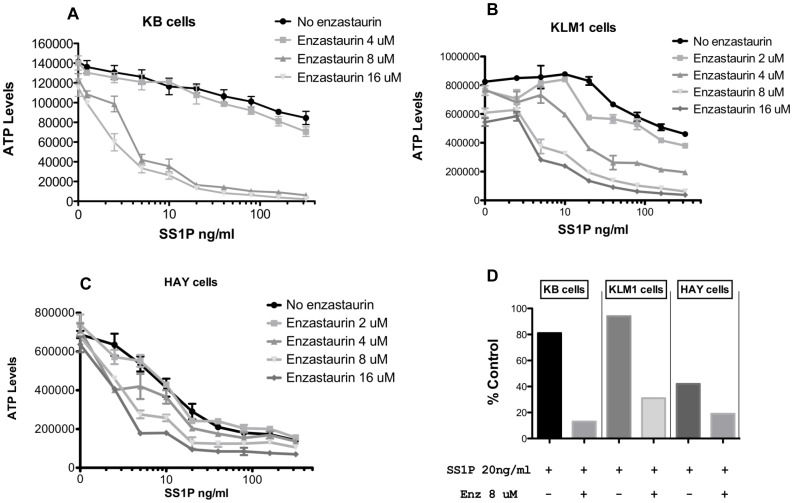
Cytotoxic activity of SS1P in combination with enzastaurin on KB, KLM1 and HAY cells. Each cell line (panel A–C) was treated with SS1P (0,20,40,80,160 and 320 ng/ml) in combination with enzastaurin (0,2, 4,8,16 uM) for 48 hrs and the viability was measured by CellTiter-Glo® Assay after 48 hrs. The viability of cells is represented in relative luminescence units. Panel D replots data showing a comparison of the three lines where SS1P at 20 ng/ml was added without or with enzastaurin at 8 uM. Data are presented as % control compared to either untreated (for SS1P) or to enzastaurin alone at 8 uM (for the combination).

A matrix of enzastaurin and immunotoxin concentrations was arrayed to determine the fold-enhancement of combination treatments versus immunotoxin alone. Enzastaurin at 4 uM exhibited a small (∼2-fold) enhancement of SS1P activity toward KB cells (Fig. 2A) while the presence of 8 or 16 uM enzastaurin enhanced killing by approximately100-fold (Fig. 2A). KB cells are partially resistant to SS1P with an IC50 in the range of 200–300 ng/ml. However, in the presence of enzastaurin, at concentrations above 5 uM, IC50s of 2–5 ng/ml were achieved. To test the general usefulness of enzastaurin, a similar matrix was organized for two other mesothelin-positive cell lines. KLM1 cells, a pancreatic cancer line, also exhibited resistance to SS1P, with an IC50 greater than 100 ng/ml. As with KB cells, combination treatments with enzastaurin sensitized cells in a dose responsive manner. At 2 uM enzastaurin there was a modest increase in sensitivity. However, beginning at 4 uM and then at 8 and 16 uM there was a substantial increase in immunotoxin sensitivity producing IC50s of 5–10 ng/ml of immunotoxin. HAY cells are more sensitive to SS1P with an IC50 of approximately 2 ng/ml - which was enhanced maximally to 0.5 ng/ml (a four-fold enhancement) with 8–16 uM enzastaurin. To determine relative IC50 values, data were normalized where each concentration of enzastaurin was considered ‘100% of control’ and % inhibition for each concentration of immunotoxin was then calculated from the resulting graphs (Figure S1 and Table 1). For comparative purposes, Fig. 2D summarizes the cytotoxic effect of a single concentration of SS1P (20 ng/ml) in the presence or absence of enzastaurin (8 uM) for the three cell lines (KB, KLM1 and HAY). The figure shows a major enhancement of SS1P toxicity, especially on cells (KB and KLM1) that exhibit partial resistance to the immunotoxin alone.

**Table 1:** IC50 of combination (SS1P & Enzastaurin) treatment relative to SS1P alone.

Cell Line	IC50 of Combination(ng/ml)	IC50 of SS1P alone(ng/ml)
KB-31	2–5	200–300
KLM1	5–10	>100
HAY	0.5	2

### Caspase Activation is Enhanced with SS1P-enzastaurin Combinations

**Figure 3:**
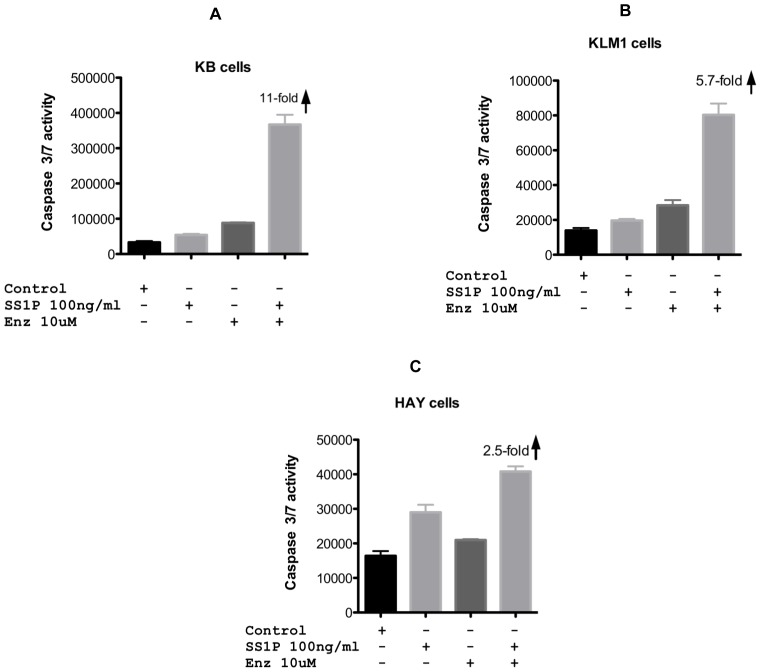
Caspase 3/7 activity following SS1P, enzastaurin or combinations of both agents. SS1P (100 ng/ml) or enzastaurin (10 uM) were added to KB, KLM1 or HAY cell lines individually or in combination. Caspase 3/7 activity is represented as relative luminescence units and was measured after cells were treated with an overnight incubation (approximately 20 hr).

The CellTiterGlo assay detects cellular ATP levels and reports on the energy status of the cell. To determine if combinations of SS1P and enzastaurin enhanced apoptosis we conducted similar combination experiments and assayed for caspase activation. For each cell line, immunotoxin-mediated apoptosis was enhanced in the presence of 10 uM enzastaurin. In the SS1P resistant lines, enhancement of apoptosis was 5–11-fold, for KLM1 and KB cells respectively (Fig. 3A and B). Enhancement of caspase activation of HAY cells was less substantial, with only a 2.5-fold increase (Fig. 3C). From these results we conclude that combining SS1P with enzastaurin leads to greater cell death.

### Enzastaurin in Combination with Agents that Inhibit Protein Synthesis

**Figure 4:**
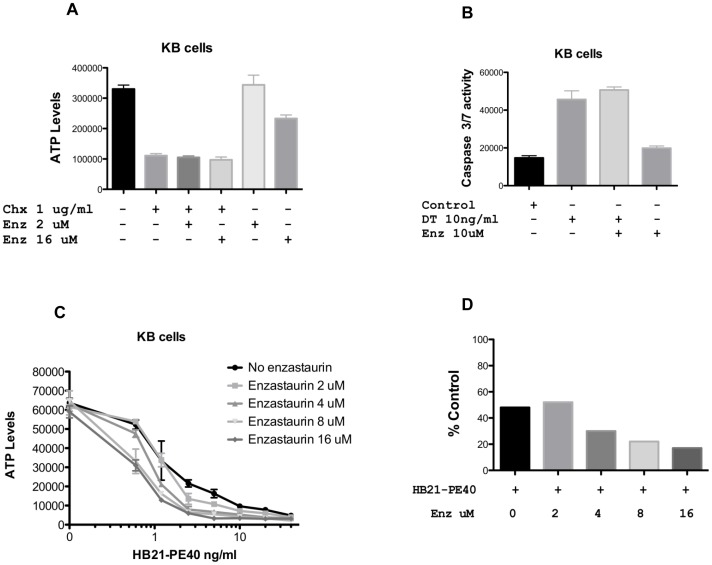
Enzastaurin in combination with agents that inhibit protein synthesis. KB cells were treated with CHX (A), DT (B) or HB21-PE40 (C) alone or in combination with enzastaurin at the concentrations indicated for 24 hrs (Caspase Assay) or 48 hrs (CellTiter-Glo® Assay). Both ATP and Caspase levels are represented in relative luminescence units. Panel D shows a replot of data where HB21-PE40 was added at 1.25 ng/ml without or with enzastaurin at the indicated concentrations. Data are presented as % control compared to untreated cells (for SS1P) or to enzastaurin alone (for the combination).

To determine if enzastaurin was a working at the level of protein translation, we incubated cells with three agents that inhibit protein synthesis. Cycloheximide reduced ATP levels at 1 and 5 ug/ml (only the 1 ug/ml concentration is shown) but there was no enhancement in the presence of either 2 or 16 uM enzastaurin (Fig. 4A). Because diphtheria toxin was so potent at reducing ATP levels, we measured caspase activation. While diphtheria toxin by itself caused a 3-fold increase in caspase, there was no enhancement with the addition of either 1 (not shown) or 10 uM enzastaurin (Fig. 4B). HB21-PE40 is a PE-based immunotoxin directed to the transferrin receptor. In contrast to DT and cycloheximide, its activity was enhanced ∼4-fold by enzastaurin concentrations in the 8–16 uM range. When plotting a single concentration of HB21-PE40 (1.25 ng/ml), with and without enzastaurin (0, 2, 4, 8, and 16 uM) the trend of enhanced cytoxicity was again evident (Fig. 4D). Thus, we confirm that enzastaurin exhibits a preference for enhancing PE-based immunotoxins, an effect which is most prominent at 8 and 16 uM.

### Mechanistic Insights

**Figure 5:**
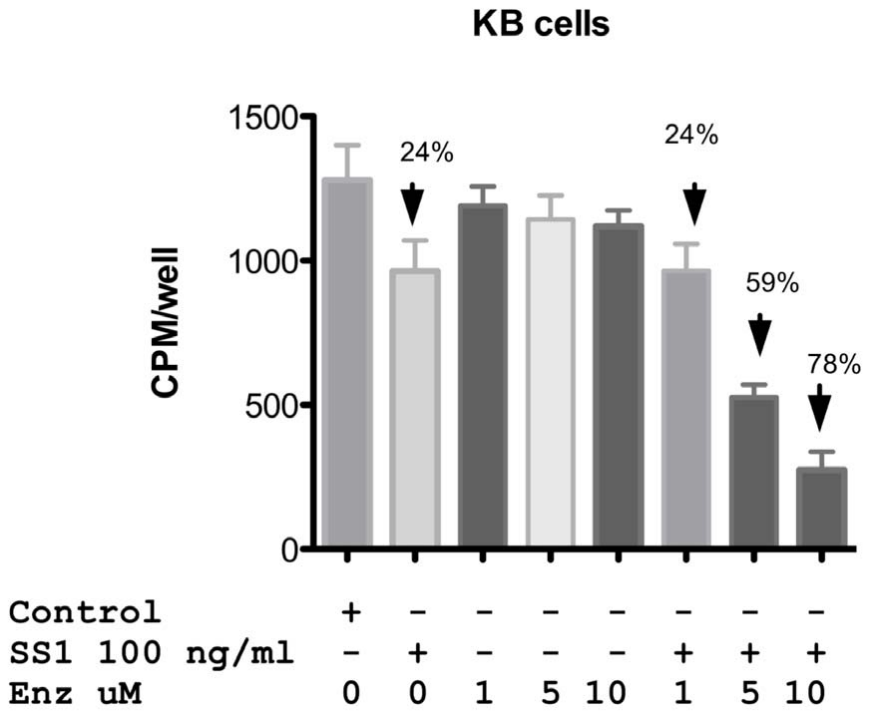
Protein synthesis levels in KB cells treated with SS1P,enzastaurin alone or a combination of both. KB cells were treated with SS1P and enzastaurin alone or in combination at the concentrations indicated in the figure. Protein synthesis was determined by measuring the incorporation of 3H-leucine into cells at 24 h post treatment. Data is represented in cpm/well. Error bars show one standard deviation of triplicate samples.

Designating a combination treatment as ‘synergistic’ implies that both compounds enhance each other’s action. Immunotoxins inhibit protein synthesis leading to the loss of Mcl-1. Inhibition of PKC can lead to the inactivation of AKT resulting in the re-activation of GSK beta that results in the phosphorylation and degradation of Mcl-1. Thus Mcl-1 is a common, albeit indirect target of both compounds. To examine the roles of immunotoxin and enzastaurin in protein synthesis inhibition, we incubated KB cells with single agents or combinations and measured the incorporation of 3H-leucine into cellular proteins. SS1P at 100 ng/ml reduced protein synthesis by approximately 25% Fig. 5. In the presence of 1 uM enzastaurin (below the synergy threshold) there was also a 25% inhibition of protein synthesis. However, this increased to 60 and 80% inhibition respectively with 5 and 10 uM combinations Fig. 5. Thus, the synergy seen with growth inhibition was also noted in protein synthesis assays.

**Figure 6:**
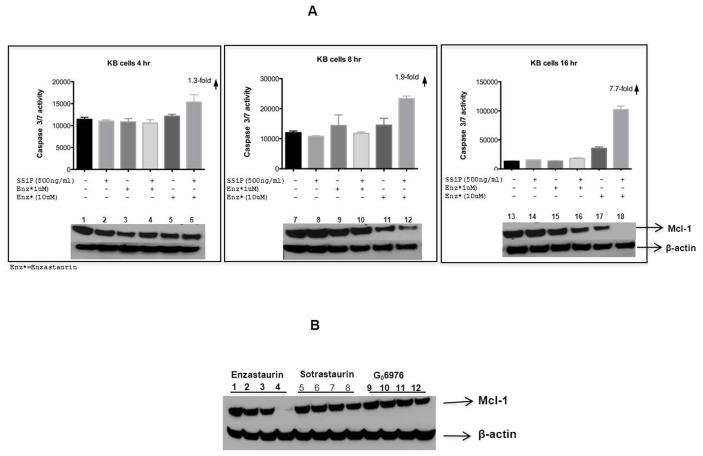
Early activation of caspase was coincident with loss of Mcl-1. (A) SS1P (500 ng/ml) or Enzastaurin (1 uM and 10 uM), were added to the KB cell line, individually or in combination. Caspase 3/7 activity was measured at time points from 4–16 h. Immunoblot analysis for Mcl-1 levels was performed as indicated in materials and methods. The concentartion of each agent is indicated in the figure. (B) Enzastaurin (10 uM) but not Sortastaurin (10 uM) or G_0_6976 (10 uM) in combination with SS1P (500 ng/ml) resulted in loss of Mcl-1 in KB cells treated overnight. Treatments were as follows: control (Lanes 1, 5 and 9), SS1P 500 ng/ml (Lanes 2,6,10), Enzastaurin 10 uM (Lane 3), SS1P 500 ng/ml+Enzastaurin 10 uM (Lane 4), Sotrastaurin 10 uM (Lane 7), SS1P 500 ng/ml+Sotrastaurin 10 uM (Lane 8), G_0_ 6976 10 uM (Lane 11) and SS1P 500 ng/ml+G_0_6976 10 uM (Lane 12).

Further, in a time course we noted that substantial activation of caspase coincided with loss of Mcl-1. Little activation was noted when enzastaurin and SS1P were combined at 4 and 8 hrs. Only at 16 hrs did combinations of 500 ng/ml of SS1P and enzastaurin at 10 uM result in ∼ 8-fold increase in caspase and complete loss of Mcl-1 (Fig. 6).

**Figure 7:**
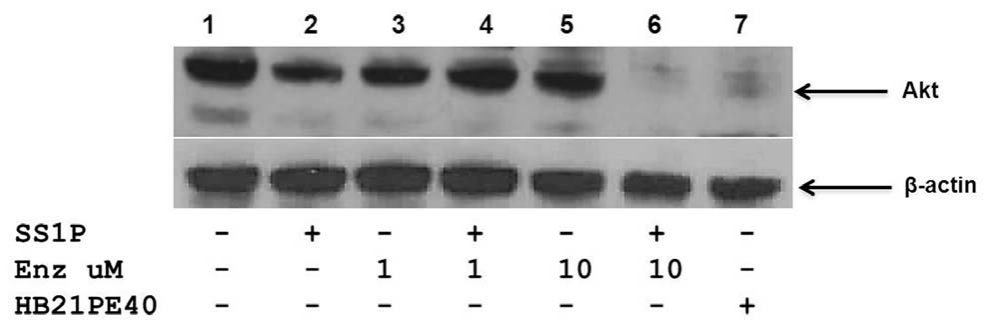
SS1P in combination with enzastaurin results in loss of Akt. KB cells were treated with SS1P, enzastaurin, SS1P+Enzastaurin, HB21-PE40 - overnight. Akt was detected via immunoblot as indicated in materials and methods. β-actin was used as a loading control. Control (Lane 1), SS1P 500 ng/ml (Lane 2), Enzastaurin 1 uM (Lane 3), SS1P 500 ng/ml+Enzastaurin 1 uM (Lane 4), Enzastaurin 10 uM (Lane 5), SS1P 500 ng/ml+Enzastaurin 10 uM (Lane 6) and HB21-PE40 200 ng/ml (Lane 7).

In searching for a mechanism to explain the loss of Mcl-1 we considered that enzastaurin was interacting with the immunotoxin pathway to make it more effective, which subsequently resulted in the loss of Mcl-1. Our early data showing that other PKC inhibitors did not produce the same level of immunotoxin enhancement led to a comparison among these agents and a determination of cellular Mcl-1 levels. We noted that only enzastaurin and not sotrastaurin or Go6976, when combined with SS1P, resulted in a complete loss of Mcl-1. Thus, there was specificity to enzastaurin action. In searching for a particular action of enzastaurin in the loss of Mcl-1, we considered the upstream pathway and examined AKT levels. AKT is known to positively regulate Mcl-1 levels [33], [34], [35]. We noted a complete loss of AKT only in the combination treatment with 10 uM enzastaurin or with very high concentrations of the active immunotoxin targeting the transferrin receptor (Fig. 7). Loss of AKT would therefore result in a more extensive loss of Mcl-1. Thus a possible explanation for the synergy with high (above 5 uM) concentrations of enzastaurin could reside in the increased delivery of toxin to cell cytosol leading to loss of AKT and the subsequent complete depletion of Mcl-1.

### Killing of Cells

**Figure 8:**
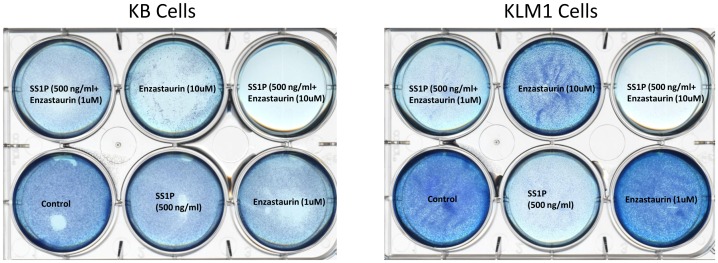
Cell viability following incubation with SS1P, enzastaurin and combinations of both in KB and KLM1 cells. KB and KLM1 cells were seeded in 6 well plates and treated with the indicated concentration of SS1P, Enzastaurin or both for 48**.**

Using cytotoxicity assays that detect energy stores, mitochondrial ‘health’ and the activation of the apoptotic pathway we have shown synergistic action of SS1P and enzastaurin. In addition here we report the loss of growth on plastic culture dishes (Fig. 8). Cells grown on 6-well plates were stained with methylene blue to detect residual cells following single or combination treatments. Only in cells (KB or KLM1 - the two most resistant lines) when treated with SS1P and enzastaurin at 10 uM was there complete loss of cells from the surface of culture dishes (Fig. 8). SS1P alone clearly resulted in less robust growth but cells were still present uniformly on the dish. And the addition of enzastaurin at 10 uM alone resulted in reduced growth but, like SS1P, there were still substantial numbers of stained cells. There was little or no loss of cells treated with enzastaurin at 1 uM. Thus, in all conditions tested, there was complete killing only when combinations of enzastaurin (above 5 uM) and SS1P were applied to mesothelin-expressing cell lines (Fig. 8).

## Discussion

Inhibiting PKC is one of many potential strategies to improve cancer treatment outcomes [2], [36]. The family of PKC enzymes comprises many isoforms, some of which contribute variously to survival, proliferation and angiogenesis. Therefore it is likely that inhibitors of PKC will have a variable beneficial action depending on the cancer cell type and the activity being targeted. Recently, we became aware that immunotoxin treatment was not universally lethal despite toxin-mediated inhibition of protein synthesis [13]. This suggests the existence of resistance mechanisms that have not been extensively reported or understood. At a practical level, the combination of immunotoxins and agents that enhance killing activity may have two benefits: it will improve our basic understanding of toxin pathways and lead to improved treatment outcomes.

Initially, we surveyed three PKC inhibitors as agents that could be combined with PE-based immunotoxins. Two of these, sotrastaurin and Go6976, exhibited some antagonism, whereby immunotoxin treatment rendered these inhibitors less potent (Fig. 1 B and C). However, the third agent, enzastaurin enhanced immunotoxin action (Fig. 1A and Fig. 2A,B&C). Enhancement was most evident in cells that exhibited partial resistance to immunotoxin treatment. Further, enhancement was concentration-dependent and evident minimally at ∼4 uM and maximally at ∼10 uM. In three-of-three cell lines, enzastaurin by itself was only ‘slightly’ cytotoxic. At a concentration of 10 uM, enzastaurin alone caused no more than a 15% reduction in ATP levels for KB and HAY cells. High concentrations of enzastaurin were cytotoxic for KLM1 cells with a 25% reduction in ATP levels (Fig. 2B). However, enzastaurin at 4 uM, which was non-toxic, still enhanced immunotoxin action in KLM1 cells by 10-fold.

Clearly, the inhibition of PKC, by itself, does not cause an enhancement of immunotoxin action. We know this because sotrastaurin and Go6976 do not synergize with immunotoxin action. In fact there was evidence of antagonism - see Fig. 1 B and C. Thus, enzastaurin must exhibit an additional cellular activity, presumably functioning as an inhibitor of another kinase or kinases. In support of this, enzastaurin is known is to be a multi-kinase inhibitor [37], [38]. With regard to enhancement of immunotoxin action, what is not clear is the nature of this additional inhibitory action and whether or not it is additive with PKC inhibition or a ‘stand alone’ inhibition. Additional experimentation will be needed to address this issue.

Enhancement by compounds that are, by themselves, non-toxic constitutes de facto ‘synergy’. Therefore, for each of the three cell lines tested, KB, KLM1 and HAY, enzastaurin and SS1P exhibited synergistic cell killing. Killing included reductions in ATP levels, caspase activation and loss of attachment from culture dishes. Investigations of mechanism revealed several features. First, both agents in combination resulted in greater reductions in protein synthesis than immunotoxin alone. Further, enzastaurin but not sotrastaurin or Go6976 in combination with immunotoxins resulted in complete loss of the prosurvival protein Mcl-1. Loss of Mcl-1 is considered pivotal in many models of cell death and in some previous reports of immunotoxin action [27], [35], [39]. Finally, there was loss of AKT protein only in two instances: high concentrations of an active immunotoxin directed to the transferrin receptor and combination treatments with enzastaurin at concentrations that produced synergistic killing.

It is unlikely that concentrations of enzastaurin at 10–16 uM can be achieved in patients with mesothelin-positive tumors. However, as mentioned above, like many kinase inhibitors, enzastaurin is known to be a multi-kinase inhibitor [37]. Thus the problem of which specific kinase(s) needs to be inhibited to produce synergistic killing is tractable and further investigations should point to key enzyme(s) that currently contribute to immunotoxin resistance.

## Supporting Information

Figure S1 **To determine the relative IC50 values for KB(A), KLM1(B) and HAY cells (C) the Cell Viability (Cell-Titer Glo) data were normalized where each concentration of enzastaurin was considered ‘100% of control’ and % inhibition for each concentration of immunotoxin was then calculated from the resulting graphs.**
(PPTX)
