# Supplementary figures and images for: Combination Treatments with the PKC Inhibitor, Enzastaurin, Enhance the Cytotoxicity of the Anti-Mesothelin Immunotoxin, SS1P
Source: PLoS One. 2013 Oct 9;8(10):e75576. doi: 10.1371/journal.pone.0075576 (PMC3794001; doi:10.1371/journal.pone.0075576)

## Slide 1
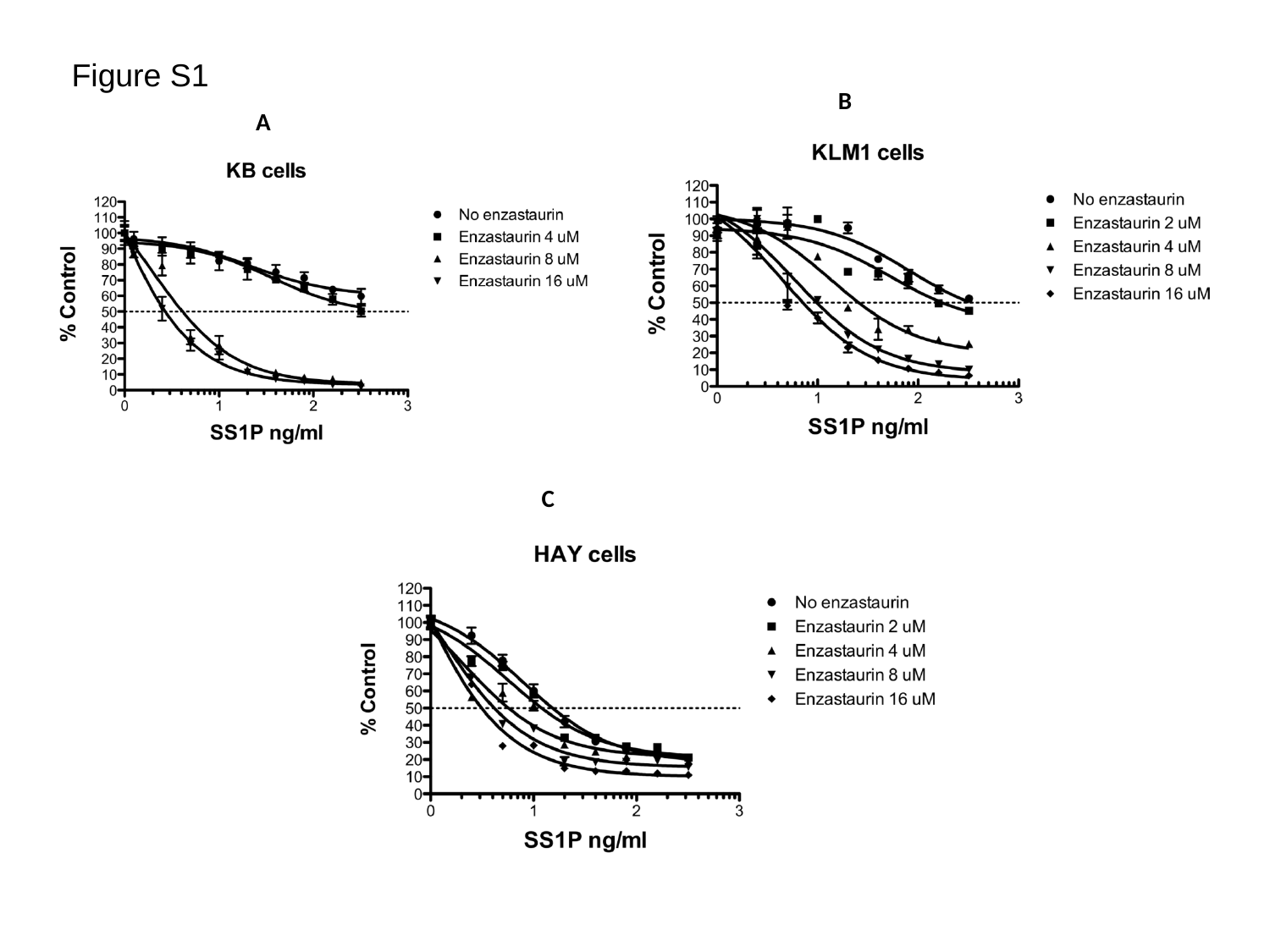

Figure S1
B
A
C

Supplement: Figure S1 — To determine the relative IC50 values for KB(A), KLM1(B) and HAY cells (C) the Cell Viability (Cell-Titer Glo) data were normalized where each concentration of enzastaurin was considered ‘100% of control’ and % inhibition for each concentration of immunotoxin was then calculated from the resulting graphs. (PPTX) [file pone.0075576.s001.pptx]
